# Neuroreceptor kinetics in rats repeatedly exposed to quinpirole as a model for OCD

**DOI:** 10.1371/journal.pone.0213313

**Published:** 2019-03-07

**Authors:** Stijn Servaes, Dorien Glorie, Sigrid Stroobants, Steven Staelens

**Affiliations:** 1 Molecular Imaging Center Antwerp (MICA), University of Antwerp, Wilrijk, Antwerp, Belgium; 2 Department of Nuclear Medicine, University Hospital Antwerp, Edegem, Antwerp, Belgium; University of Kentucky, UNITED STATES

## Abstract

**Background:**

Obsessive-compulsive disorder (OCD) is a chronic, incapacitating, early onset psychiatric disorder that is characterized by obsessions and compulsions originating from a disturbance in the cortico-striato-thalamico-cortical circuit. We implemented the preclinical quinpirole (QP) rat model for compulsive checking in OCD to analyse the behaviour and visualize the D2R, mGluR5 and GLT1 density in order to contribute to the understanding of the neuroreceptor kinetics.

**Methods:**

Animals (n = 14) were exposed to either saline (1 mL/kg) or QP (dopamine D2-agonist, 0.5 mg/kg) twice-weekly during 7 weeks. After each injection animals were placed on an open field test. After model setup, animals were placed in a behavioural cage equipped with tracking software and hardware in order to analyse the behaviour. Subsequently, sagittal slides were made of the CP in the right hemisphere and a staining was done with the D2R, mGluR5 and GLT-1 antibody to visualize the corresponding receptor.

**Results:**

The QP animals displayed a strong increase in travelled distance (+596.70%) and in the number of homebase visits (+1222.90%) compared to the control animals. After chronic exposure to QP, animals had a significantly (p < 0.05) higher percentage of D2R density in the CP (7.92% ± 0.48%) versus 6.66% ± 0.28% in animals treated with saline. There were no differences for mGluR5 and GLT1 receptor density.

**Conclusions:**

Chronic exposure to QP leads to hyperlocomotion and an increase in D2R density. Furthermore, as mGluR5 and GLT1 density did not seem to be directly affected, decreased levels of glutamate might have influenced the binding potential in earlier reports.

## Introduction

Obsessive-compulsive disorder (OCD) is a psychiatric disorder that is characterized by distressing, impairing obsessions or compulsions consisting out of repetitive behaviours and thoughts [1]. Often these alternate in a cyclic manner and are accompanied by certain types of avoidance behaviour to reduce the stress induced by obsessions, ultimately reinforcing the obsession [2,3], resulting in a significant reduced quality of life for both the patient as well as their close relatives [4]. With a lifetime prevalence for OCD among adults in the United States estimated between 1% to 3% [5], it is ranked in the top 10 leading causes of disability [6].

It is believed that disturbances of the cortico-striato-thalamico-cortical (CSTC) circuitry [3,7,8] are at the basis of this disorder. Nonetheless, the exact underlying pathophysiology of OCD is still poorly understood. It was initially suggested that an altered local neurotransmission in this CSTC loop causes a certain behavioural dysinhibition [9]. A number of neurotransmitters are involved in this circuit. Originally, the manifestation of OCD was considered to be a serotonergic (5-HT) disorder [10]. However, as selective 5-HT reuptake inhibitors (SSRIs) have proven to be ineffective, the role for 5-HT has diminished over the past recent years [11]. There being a strong connection between 5-HT and dopamine (DA) [12] and a significant improvement of the symptoms after treatment with DA agonists [10,11], it was a logical step to consider DA as a candidate for the cause of the pathology. With DA being released from the nigral projections into the striatum, there is an activation of the direct pathway, which ultimately results in disinhibition of the thalamus with stronger projections running to the cortex. On the other hand, when DA is absent, the indirect pathway takes over, inhibiting the thalamus and causing less signalling to the cortex [8]. A disturbance in this circuit could therefore give rise to an uncontrolled feedback loop, resulting in the compulsive behaviour. The particular role that DA has in this loop, and the implications of this loop in OCD, gave rise to a DA-hypothesis. However, as approximately one in three patients do not improve significantly with current treatment modalities even after optimization [13], it is clear that the research frame should be expanded beyond these two neurotransmitters.

As of recent, another neurotransmitter, glutamate, has risen to the surface in OCD-related research [14]. Evidence for this came from the modulation of the N-methyl-D-aspartate receptors (NMDAR), as it significantly reduced the compulsive behaviour, both in animal models [15] and in humans [16]. Furthermore, our own group has previously highlighted a glutamatergic dysfunction in a preclinical model for OCD [17]. Another PET study in humans found a significant positive correlation between the occupancy of the metabotropic glutamate-5-receptor (mGluR5) and the actual presence of obsessions [18]. Additionally, compulsive behaviour in mice was reduced by administering the mGluR5 antagonist 2-methyl-6-phenylethyl-pyrididine (MPEP) [19]. Together with the positive influence of a memantine augmentation to the SSRI treatment [16], this opened up an entirely new line of glutamate-modelling treatment options [13,20]. All this hints towards direct involvement of glutamate and the glutamate receptors in the manifestation of the disease.

As there is a lack of patients that are naïve for followed therapies or previous medication, clinical OCD research is often difficult to interpret. Furthermore, the heterogeneity due to intrinsic differences in the individual manifestation of OCD itself, and the comorbidity with other psychiatric disorders, complicate cases even further. Therefore, preclinical models are an excellent tool to focus on the underlying pathophysiological mechanisms of OCD. A model that is commonly used in preclinical research is the quinpirole (QP) sensitisation rat model for OCD, which was originally developed by Szechtman et al. in 1998 [21]. This model is set up by chronically injecting rats with the D2 agonist QP. After each of these injections, animals are then placed on a 160 x 160 cm table with 4 different objects for a 30-minute lasting open field test (OFT). Compared to the animals that received saline, the animals receiving QP have a significant increase in locomotion and visit one or two of the objects excessively [21]. It is believed that the continuous administration of the D2-agonist causes a sensitisation of the D2-receptors, which in turn causes a disruption in the CSTC-circuit, ultimately resulting in the compulsive-like behaviour. Also it appears that the animals display particular environmental-dependent behavioural patterns around the objects, which strongly resembles the human condition [22–24]. The research that followed on these findings mainly focused on the behavioural side for pharmacological validation of certain drugs or treatments [25–27]. Previous work of our group has established the effects of repeated exposure to QP and the open field with molecular imaging of both the glucose metabolism [28], as well as dopamine (DA) receptor occupancy and metabotropic glutamate 5 receptor (mGluR5) distribution [17]. This has led us to design the current study to validate the previous DA and mGluR5 results in the QP model with ex-vivo immunohistochemistry extending it with the glutamate transporter 1 (GLT-1), in order to determine whether the differences are indeed a consequence of receptor downregulation or internalisation.

## Material and methods

### Animals

This study was performed in accordance with the European Communities Council Directive of November 24^th^, 1986 (86/609/EEC) for care of laboratory animals with approval of the local ethical committee (University of Antwerp under number 2014–18). All necessary efforts were made in order to reduce the number of animals and minimize suffering. Fourteen (n = 14) naïve male Sprague Dawley rats (Harlan, the Netherlands, ranging in between a minimum of 285 and a maximum of 550 g from the beginning until the end of the experiment) were housed in a temperature- and humidity-controlled vivarium in IVC-cages with a 12-hour light-dark cycle. Food and water were available ad libitum. All experiments were performed during daytime. All applicable institutional and national guidelines for the care and use of animals were followed.

### Experimental setup

Prior to the start of the experiments, all animals (n = 14) were handled during 2 minutes for 5 consecutive days in order to reduce the stress of the animals in the following experiments. Animals were then divided into a control condition (CTRL; n = 7) and an exposure condition (QP; n = 7).

A subcutaneous injection of QP (Sigma-Aldrich, St. Louis, MO, USA; 0.5 mg/kg dissolved in saline, NaCl 0.9%, at a volume of 1 mL/kg) was given twice a week for a total duration of 5 weeks to the animals of the QP group. Animals of the control group on the other hand received a subcutaneous injection of saline (1 mL/kg; NaCl 0.9%), also twice a week, for the same total duration. Fifteen minutes after each injection, the animals of each group were subjected to an Open Field Test (OFT) monitoring them for 30 minutes on a 160 x 160 cm table with 4 objects on fixed locations as described earlier (Szechtman et al, 1998). The total distance travelled, as well as the frequency of visits at the different objects, were analysed. The object with the highest number of visits was identified as the homebase (HB). Additionally, as an increased locomotion could also cause an increased frequency of visits, an algorithm was applied to compare the observed versus the expected number of returns to the HB, specified earlier by Winter [27]. This was done to evaluate the frequency independent from the travelled distance.

After model setup, animals were placed in a behavioural cage in which their behaviour was monitored for the total period of 45 minutes post injection. Activity, rotations, behaviour and locomotion were monitored and recorded for both groups. Also freezing behaviour was tracked with the mobility parameter registering the frequency that the complete area of animal did not pass a threshold (20%) set for moving per recorded sample. All data from both the open field and from the behavioural cage was checked for normality by means of a Shapiro-Wilk test. Data collected from the open field after each injection was analysed with a two-way ANOVA using the injection number and the 2 different treatments as independent variables, while the extracted parameter functioned as the dependent variable. In case data was normally distributed, an independent 2-group t-test was done to test for significance at the 0.05 alpha level. If data was not distributed normally, a Mann-Whitney-Wilcoxon test was done to establish significance at the 0.05 alpha level.

After the final behavioural experiment, animals were immediately euthanised and both of their hemispheres were stored separately in paraffin. Sagittal slides of the right hemisphere of the brain of the CP of each animal were made in duplicate with a microtome (Microm HM340E) with R35 Microtome blades at a thickness of 5 micrometres on Superfrost Plus slides (Menzel-Gläser). These slices were stored at -80°C. Staining with all antibodies was done with provided protocols that were previously validated in literature [29–31]. Before staining, slides were acclimatized at room temperature during 5 min before antigen unmasking with 10mM sodium citrate buffer pH 6.0 (2.10 g citric acid/L of H2O) at 95 degrees for 10 minutes. Hereafter slides were allowed to cool down for 20 minutes while remaining in the buffer solution. They were washed 3 times in PBS (pH 7.4) for 5 min each after which they were immersed in water containing 3% H_2_O_2_ for 15 min at room temperature to quench endogenous peroxidases. After three washes of 5 min, each in PBS, sections were preincubated with 5% normal goat serum (NGS) in PBS at room temperature for 1 h for the mGluR5 and DA staining, while 10% normal horse serum (NHS) was used for the GLT-1 staining. Then slides were incubated with polyclonal D2R primary antibody (1/50 in PBS with 5% NGS; SC-5303 Santa-Cruz) mGluR5 antibody (1/200 in PBS with 5% NGS; AB5675 Millipore) or GLT-1 antibody (1/2000 in PBS with 10% NHS; AB205248 Abcam) overnight at room temperature. Subsequently, after three 5 min rinses in PBS, the D2R sections were incubated with secondary antibody (igG2a-HRP; 1/500 in PBS with 5% NGS) for 2 hours. The mGluR5 sections were rinsed three times for 5 minutes using PBS with 0.03% Triton, before incubating them with the secondary antibody (GARb; 1/500 in PBS) for 1 hour. The GLT-1 sections were incubated for 1 hour with secondary antibody (GARb-HRP 1/500 in PBS) after three 5 min rinses in PBS. After washing all slices three times for 5 min each in PBS, the peroxidase reaction was carried out with DAB reagent (Dako) for 10 minutes. The reaction was then stopped in tap water.

All pictures were taken with a Nikon Ti wide-field microscope (Nikon Instuments, Paris, France), digitalized by Nikon Digital Sight DS-FI2 camera, using NIS Elements AR software, version 4.51.01 (Nikon Instruments) in a 9x11 matrix.

Using Image J (Image J version 1.47) the D2R, mGluR5 and GLT-1 immunoreactivity was analysed. Analysis was performed on 5 rectangles of equal size in the CP and consisted out of measuring the area fraction, which refers to the percentage of pixels in the image or selection that were coloured by the staining, following the application of a set threshold for each image to eliminate any of the background and evaluate each image in a similar manner. Statistical difference was tested by means of an unpaired t-test with Welch’s correction.

## Results

No unexpected abnormal animal behaviour was seen over the course of the experiments. Animals tolerated the administered dosage of QP well and no unexpected adverse effects were seen. After model setup, at week 6, animals of the CTRL group had a weight of 411.42 ± 14.14 g, significantly different from the QP group with 387.29 ± 13.57 g.

### Open field experiment

**Fig 1:**
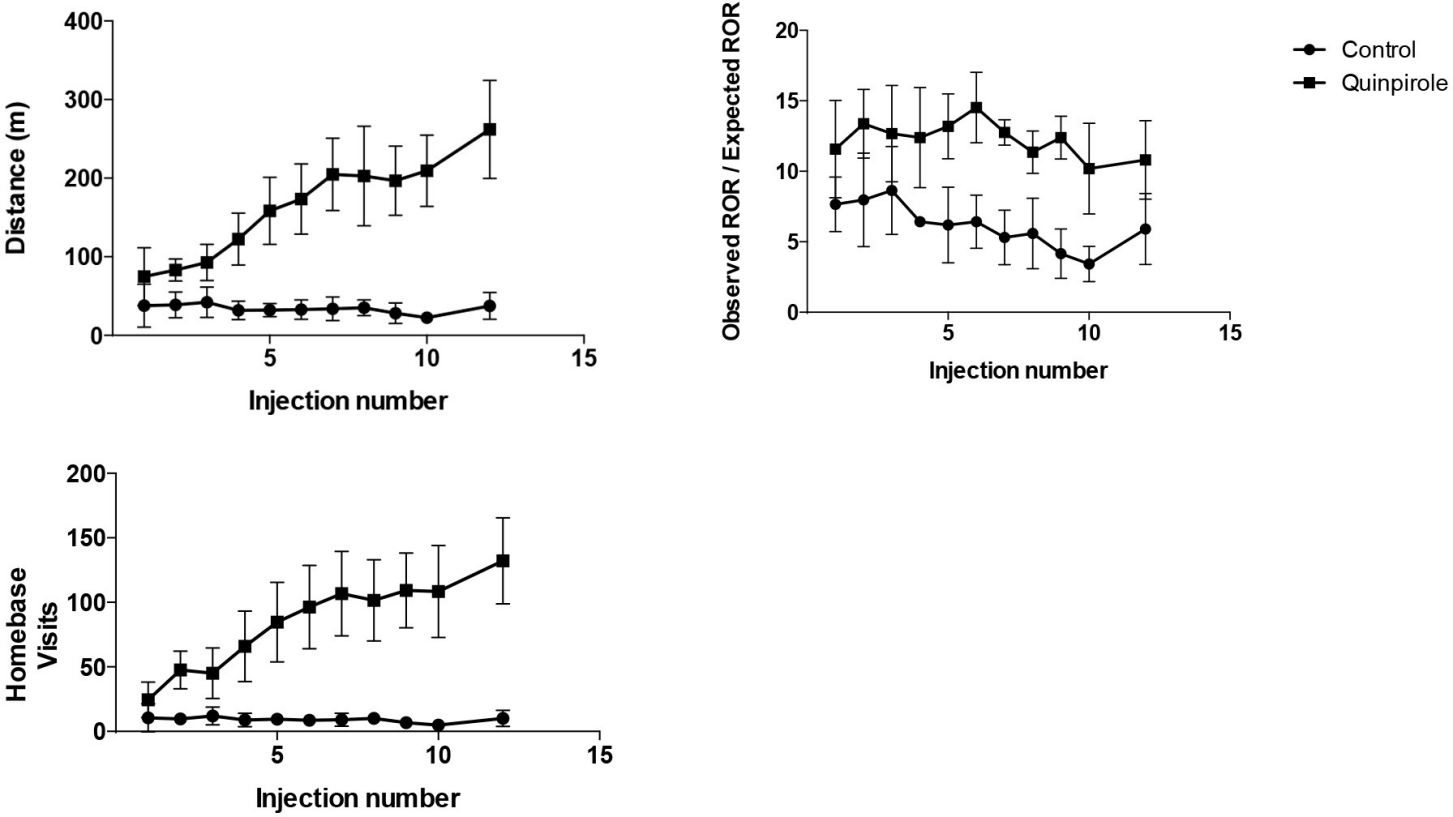
Open field test. The parameters of the Open Field Test (OFT)–distance, homebase visits and observed ROR/expected ROR–are plotted here over time. The changes on all parameters were significantly different (p < 0.05) between the two groups from injection 2 onwards. As injection 11 was combined with the cage experiment, animals were not exposed to the OFT on this day.

**Fig 2:**
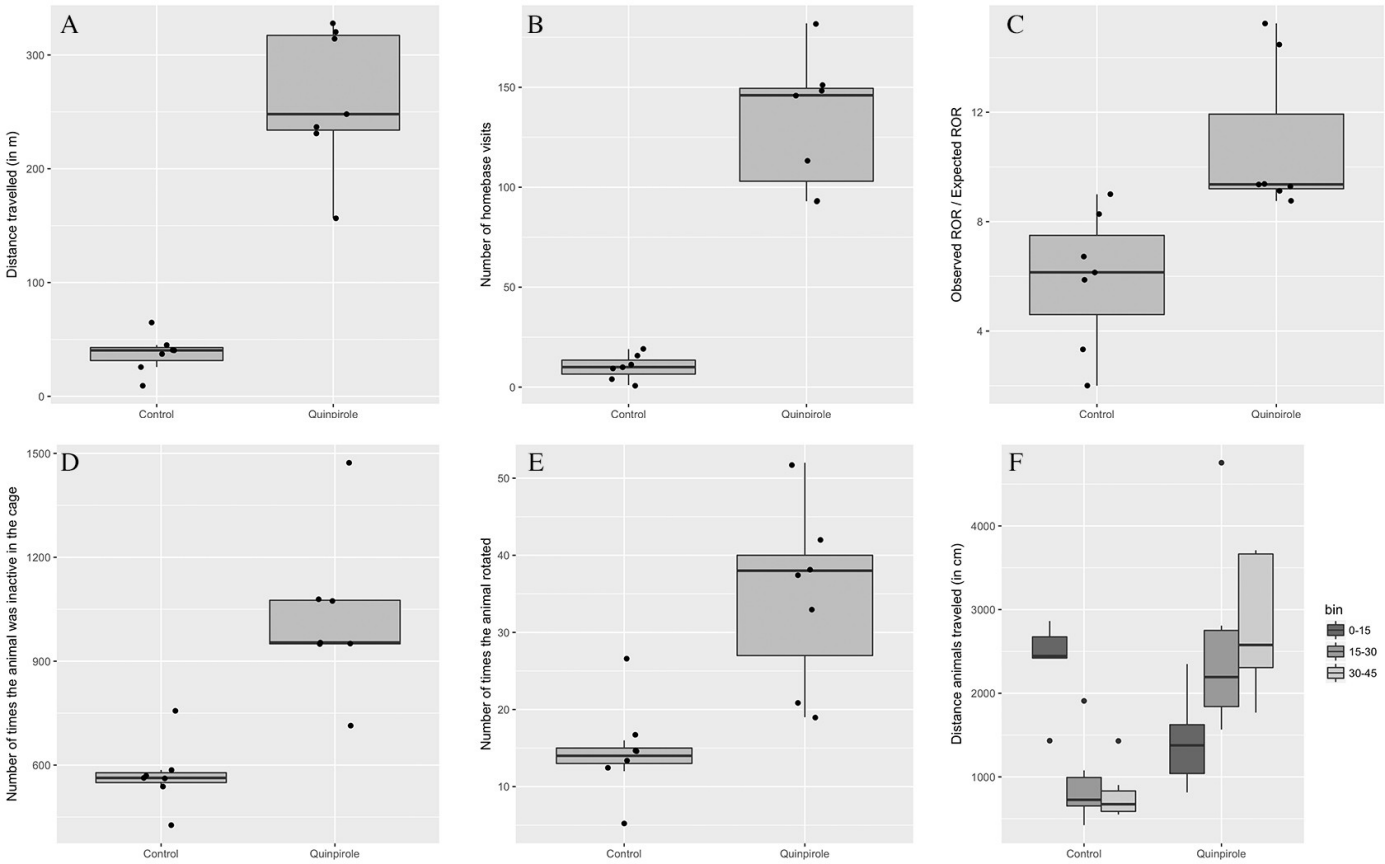
Behavioural data. The mean travelled distance (A) as well as the number of HB visits (B) are plotted here for the quinpirole (QP) condition and the control (CTRL) condition. Also, the ratio of observed to expected HB stops (C) is shown for both the chronic QP group and the chronic CTRL group. Additionally the total frequency of inactivity as a measurement for freezing behaviour (D) is and the total amount of rotations (E) that animals performed after receiving either quinpirole or saline are displayed. Finally, the activity of the animals is displayed with the total duration spent in the cage split into different time bins of each 15 minutes (F). Individual points, median and standard deviation of the group are displayed.

Immediately after injection 2, animals that were treated with QP showed an increase in the total distance travelled and in the number of HB visits (p < 0.05) as revealed by a two-way ANOVA indicating that both the injection number, QP administration and the interaction between the two had a significant on the distance travelled (Fig 1). Over time, the mean travelled distance as well as the number of HB visits increased for the QP condition when comparing to the CTRL group, resulting in a 6-fold increase (+596.70%) at the final open field test (262.10 ± 62.37 m vs 37.62 ± 17.07 m) and a 12-fold increase (+1222.90%) in the number of HB visits (132.29 ± 33.46 vs 10.00 ± 6.27) at the same follow-up time point (Fig 2A and 2B), both significant (p < 0.05) according to an independent 2-group t-test. Furthermore, a two-way ANOVA revealed that both the QP administration and the injection number, but not the interaction between the two, had a significant effect on the ratio of observed to expected HB stops. At injection 12 this was significantly (p < 0.05) higher in the chronic QP group, when compared to the chronic CTRL condition (10.80 ± 2.79 vs 5.91 ± 2.51), revealed by a Mann-Whitney-Wilcoxon test, as shown in Fig 2C.

### Behavioural cage

In the cage (15–45 min post-injection), an independent 2-group t-test, showed that animals receiving QP displayed a significant (p < 0.05) higher frequency of inactivity indicating a higher number of freezing behaviour (1027.57 ± 230.54 vs 571.71 ± 97.30) as shown in Fig 2D. Animals receiving QP also performed more rotations compared to animals receiving saline (34.71 ± 11.63 vs 14.57 ± 6.52), displayed in Fig 2E.

Furthermore, the entire duration spent in the cage was split in different time bins of 15 minutes to determine the most active phase (Fig 2F) and evaluated for significant differences by a Mann-Whitney-Wilcoxon test. For CTRL animals the initial 15 minutes contained the most activity with a distance traveled of 24.18 ± 4.78 m. The later time bins displayed little movement at all with a distance traveled of 9.07 ± 7.85 m for the second time bin and 7.85 ± 3.10 m for the third time bin. The QP animals on the other hand showed significantly (p < 0.05) less movement compared to the CTRL animals in the first time bin (14.1 ± 5.2 m), but significantly (p < 0.05) higher movement compared to the CTRL animals in the second and third time bin (25.28 ± 10.85 m and 28.57 ± 8.0717 m).

### Immunohistochemistry

**Fig 3:**
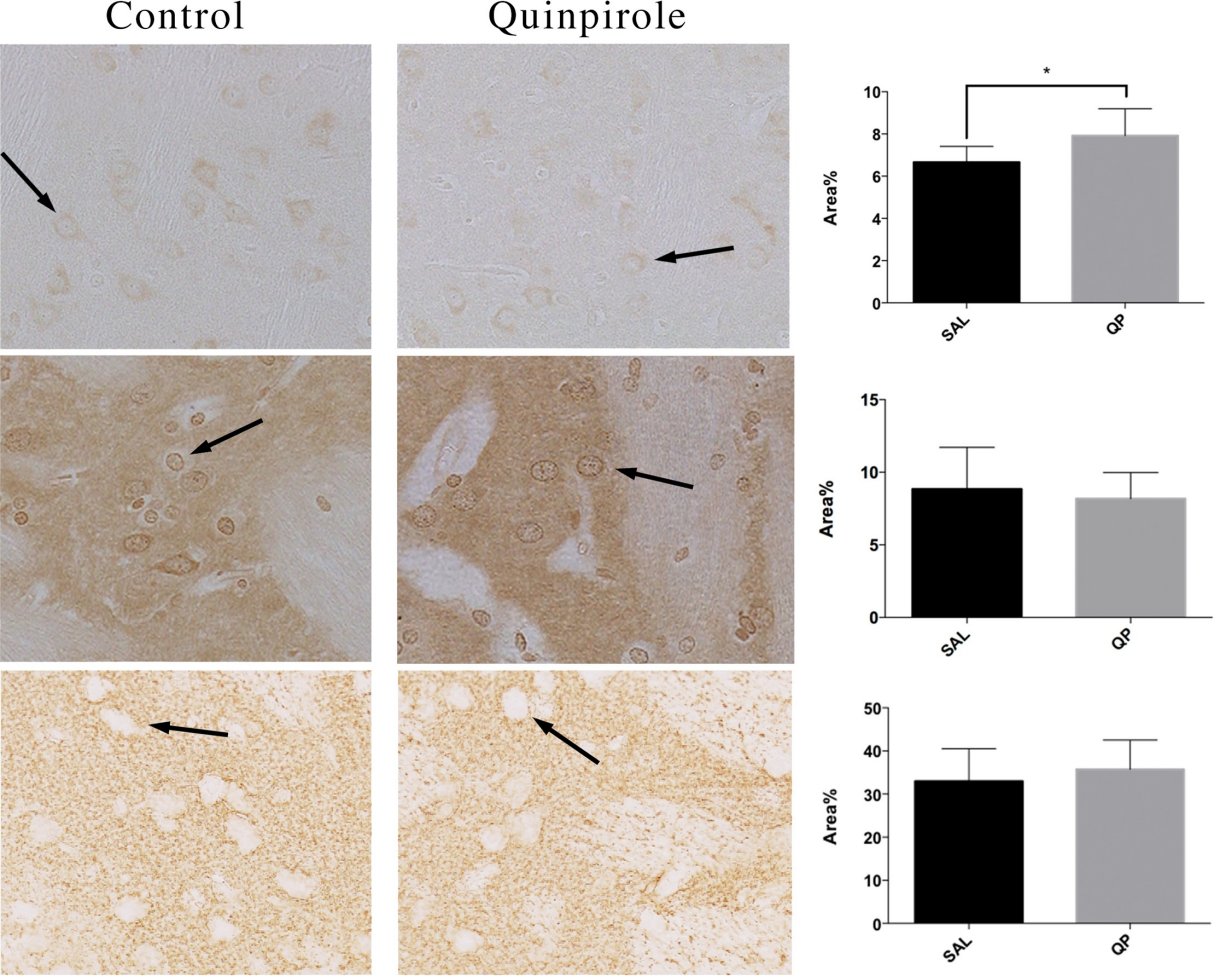
Immunohistochemistry. Image (20x) of the caudate putamen of an animal that was exposed to either chronic quinpirole (QP) or functioned as a control (SAL) after staining with either the D2R antibody (top), the mGluR5 antibody (middle) or the GLT-1 antibody (bottom). As GLT-1 is present on the astrocyte, the staining will occupy the space between neuronal cells. These neuronal cells will therefore remain uncoloured. For visual representation, one exemplary neuron is marked in each staining with an arrow. In the right utmost column, the area percentage expressed as percentage of pixels in the image that have been highlighted by the staining after either QP (n = 7) or SAL (n = 7). Individual points, median and standard deviation of each group are displayed. * p < 0.05.

Staining with SC-5303 was done successfully with a good separation of background and D2 receptors. After model setup, animals treated with QP and exposed to the OFT for the duration of the experiment, had a significant (p <0.05) higher percentage of D2 receptors highlighted in the caudate putamen (7.92% ± 0.48%) compared to animals treated with saline (6.66% ± 0.28%) as displayed in the top of Fig 3.

Staining with AB5675 clearly separated the background from the actual mGluR5 receptors. No difference in the area fraction was found when evaluating the mGluR5 staining of the QP group versus the SAL group in the CP (Fig 3 middle).

Also for GLT-1 the staining successfully separated the background from the actual GLT-1 receptors. However, there was no difference in area fraction of the staining when comparing the QP group to the SAL group in the CP (Fig 3 bottom).

## Discussion

**Fig 4:**
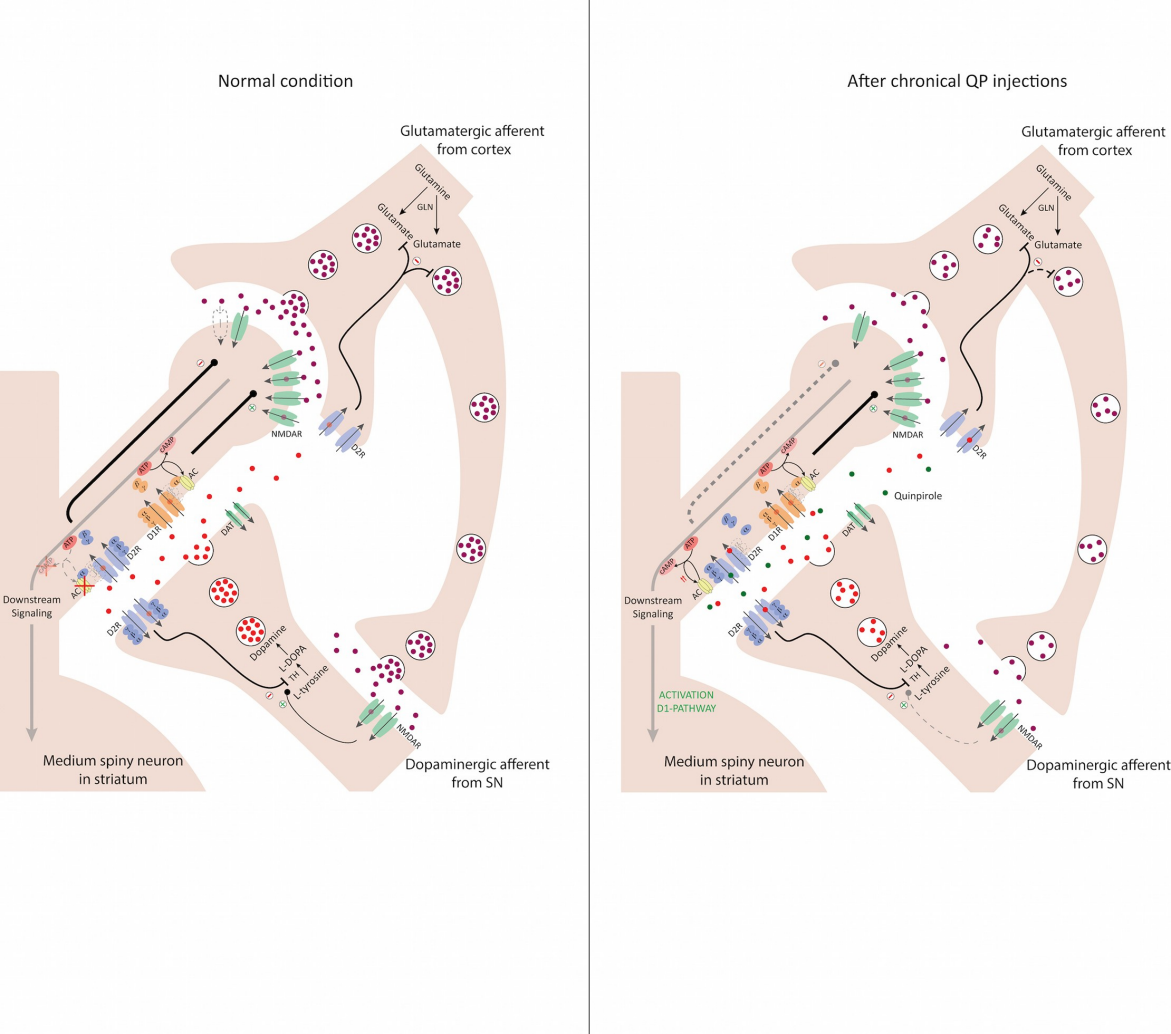
Chronic quinpirole exposure. A) The striatum consists for 95% out of medium spiny GABAergic neurons which branch out in a number of dendrites. On the heads of the dendrites, glutamatergic neurons connect that are often coming from the cortical regions. This then results in downstream signalling. This signalling can be modified and altered by DA-ergic neurons connecting on the branches. In the normal condition a balanced system is in place whereby release of DA will trigger postsynaptic D1 and D2 receptors. These G-coupled receptors will determine the amount of conversion from ATP to cAMP, a secondary messenger which affects downstream signalling. The triggering of the presynaptic D2-receptors results in a feedback mechanism by inhibition of tyrosine hydroxylase, responsible for the conversion of L-tyrosine to L-DOPA eventually leading to Dopamine. Likewise, the D2-receptors present on the glutamatergic neuron inhibit glutamate release which will reduce downstream signalling and further DA-release. DAT transporters participate in the reuptake of DA present in the synapse. B) A different scenario takes place when, due to constant stimulation of the D2-receptor, the receptor becomes sensitized. This will increase the inhibition on the release of DA as well as glutamate, leading to less release of both neurotransmitters. However, due to the constant activation of the D2R receptor a process called heterologous sensitization occurs whereby the consistent activation of Gα_i/o_-coupled receptors results in a compensatory increase in adenylyl cyclase. This will result in a D2-mediated activation of the D1-pathway resulting in the displayed motor behaviour.

This study evaluated the effect of chronic administration of QP in combination with OFT as a model for OCD on D2R, mGluR5 and GLT-1. Model setup was successful as shown by the results from the OFT. Animals from the QP group showed excessive checking behaviour when compared to animals from the SAL group (Fig 2), that gets progressively worse with continuous exposure to QP (Fig 1), in correspondence to earlier studies using the same model [17,28]. Consistent with expectations, control animals show the most activity during the first fifteen minutes in the behavioural cage. As the novelty of the environment wears off, the activity of these animals decreases, as is shown in the following 30 minutes. An opposite effect is visible for the animals treated with QP, as there is significantly less movement compared to the control group, in the first fifteen minutes, but significantly higher movement compared to the control animals in both the second and third time bin of fifteen minutes (Fig 2F). Likely this earlier ‘inhibition of movement’ is due to the stimulation of the D2R in the CP, activating a downstream cascade of interactions, known as the indirect pathway, leading to reduced activity. However, because of chronic exposure to QP, a secondary mechanism seems to override this particular cascade resulting in an uncontrolled feedback loop and hyperactivity. Likewise, as discussed previously, in clinical OCD, the underlying cause of the symptoms is thought to be a disturbance in the CSTC-circuit, leading to an uncontrolled feedback loop. Interestingly, the induced disturbance in the CSTC circuit in animals, by sensitization of the D2R through continuous exposure to QP, therefore seems to give rise to symptoms that are more D1-mediated (Fig 4). It has previously been reported by Chester et al., that repeated activation of D2-like receptors in vivo is associated with sensitisation of D1 dopamine receptor-mediated signalling in the caudate-putamen [32]. This is explained by heterologous sensitisation, whereby persistent activation of D2-coupled receptors results in a compensatory increase in adenylyl cyclase activity [33] and the resulted increase in cAMP then causes the D1-mediated signal transduction (Fig 4).

Escobar et al. reported that repeated administration of QP induced locomotor sensitisation with reduced glutamate levels coming from the prefrontal cortex together with a decreased phasic and tonic dopamine neurotransmission while conserving presynaptic D2 receptor function [34]. This decrease in dopamine release has been measured preclinically as well, by means of microdialysis, reporting a decrease of 33% in extracellular dopamine in the nucleus accumbens after chronic QP [24]. Likewise, our results show that animals exposed to the model setup had a significant increase in D2R density in the caudate putamen and locomotive behaviour, compared to the animals receiving SAL (Fig 3 top). With D2R negatively affecting dopamine release a decrease in extracellular dopamine is to be expected upon activation of the receptors. Furthermore, a significant increment in D2 receptors in high affinity state was reported after chronic QP [35,36]. Another study described this supersensitivy of D2 receptors after neonatal QP treatment [37]. Nonetheless, results have not been conclusive in this regard. Earlier reports also describe D2 receptor downregulation, when quinpirole is administered repeatedly in adulthood [38,39]. These conflicting results indicate a more complex underlying mechanism than what was initially suggested, and therefore depend on dosage, method of administration and age of the animals. With D2 located both presynaptically and postsynaptically, these factors could influence or mediate the feedback inhibition. Previous work using this QP model to investigate the D2R in-vivo distribution using [11C]-Raclopride (RAC) with PET/CT, reported significant decreases in tracer distribution after exposure to QP both acutely and chronically when compared to SAL [17]. It appears that D2Rs are in a high affinity state after chronic QP stimulation and hence bind more endogenous dopamine. Consequently, the competition for the tracer to bind to the D2R will increase and the binding potential of the PET tracer lowers [17].

Regarding glutamate, no significant changes were found in both the mGluR5 dataset (Fig 3 middle) and the GLT-1 dataset (Fig 3 bottom) of the QP-sensitized animals when comparing to the saline treated animals. Previous work using ABP-688 to visualize the mGluR5 distribution, described significant increases in the caudate putamen, the amygdala and the anterior cingulate cortex [17]. However, as this study found no changes in the caudate putamen, which is the first site of action and the region that was most affected in terms of ABP-688 binding potential, this is likely the case as well for the lesser affected regions downstream from the caudate putamen. With our IHC data showing no significant difference in mGluR5 density between the two groups, this therefore indicates that the earlier reported changes in binding potential have a different driver. As previously shown by Zimmer et al. [40] the affinity of the mGluR5 receptor for the tracer [11C]ABP-688 could be positively affected by a decrease in glutamate. Indeed, with QP being a D2R-agonist and glutamate release being regulated by D2R, the inhibition caused by D2R-activation results in a decrease in glutamate release [34] (Fig 4), increasing the affinity of the receptor for the tracer, which confirms the visualized increases in ABP binding potential [17] without receptor density chances. Further evidence for this theory comes from the GLT-1 IHC data, showing no difference in GLT-1 expression. This underlines that a decrease in glutamate comes from an inhibition of release (Fig 4) rather than an increased reuptake into the astrocytic compartment.

In conclusion, this study revealed an increase in D2R density after chronic exposure to QP, which might be caused by the interplay between the D2R high affinity state causing heterologous sensitisation resulting in D1-mediated increase in locomotion in concert with decreased dopamine and glutamate release.

## Supporting information

S1 File Behavioural cage data. (XLSX)

S2 File Open field data. (XLSX)

S3 File D2R data. (CSV)

S4 File GLT1 data. (CSV)

S5 File mGluR5 data. (XLSX)
